# Pragmatic functions of evidentiality in diplomatic discourse: Toward a new analytical framework

**DOI:** 10.3389/fpsyg.2022.1019359

**Published:** 2022-12-01

**Authors:** Zhongyi Xu

**Affiliations:** Department of Foreign Languages, Shaoxing University, Shaoxing, Zhejiang, China

**Keywords:** evidentiality, diplomatic discourse, analytical framework, pragmatic functions, normalization of ideology, legitimation

## Abstract

This paper examines the pragmatic functions of evidentiality categories in diplomatic discourse by illustrating a new classification of English evidentiality. It adopts a data-based approach by analyzing a corpus of thirty English political speeches from three US presidents (including Bush, Obama, and Trump). The results show that: (i) Evidentiality can be classified into three categories: personal sources; shared sources and other sources. (ii) Besides the function of (de)legitimation, evidentiality can also be used to normalize the speaker’s ideology. (iii) Shared sources of evidentials reflect the speaker’s ideological bias, because they encode the speaker’s presupposition of authority, facts, or shared knowledge. (iv) Personal sources of evidentials mean that the speaker is more willing to take verbal responsibility. (v) Other sources of evidentials reflect the speaker’s lower responsibility for the information he/she offered. (vi) The use of the three evidential sources reflects the speakers’ different responsibilities for their propositions and reveals their subjective or intersubjective stance.

## Introduction

Evidentiality has been studied widely since Boas (1938) first discussed it in linguistic studies. Traditionally, evidentiality can be referred to as “sources of information” (e.g., Jakobson, 1957; Willett, 1988) or “source of evidence” (DeLancey, 2001, p. 369) or “the linguistic coding of source and reliability of information” (Mushin, 2000, p. 927) or “evaluation of evidence” (Portner, 2009, p. 263). In addition, evidentiality can also indicate the speaker’s cognitive justification for his or her propositions (Boye and Harder, 2009). Indeed, the linguistic domain of evidentiality not only allows speakers to mark their source of knowledge (information) but also shows how they assess this knowledge in terms of reliability and reveals the speakers’ communicative purposes with regard to its pragmatic functions.

The previous studies of evidentiality, however, have focused on “its formal or semantic properties in grammaticalized systems” (Mushin, 2000, p. 927), while few researchers focus their attention on the pragmatic functions of evidentiality in specific discourse contexts (e.g., Mushin, 2000; Hart, 2011; Marín-Arrese, 2011b; Reber, 2014; see “background: evidentiality in discourse studies” for details).

Evidential types^1^ have also been a subject of comprehensive investigation from different perspectives (e.g., Anderson, 1986; Chafe, 1986; Willett, 1988; Nuyts, 1992; De Haan, 1999; DeLancey, 2001; Plungian, 2001; Aikhenvald, 2004; Bednarek, 2006; Squartini, 2008; Whitt, 2010; Hart, 2011; Marín-Arrese, 2011a,b; Mushin, 2013). For example, Willett (1988, p. 57) divided evidentials into “direct” and “indirect” evidence. Based on Willett’s (1988) evidential types, Plungian (2001, p. 352) classified indirect evidence into two types: “reflected” and “mediated” evidence. Chafe (1986, p. 263), however, proposed a broader category of evidentials, including “source of knowledge,” “mode of knowing,” and “knowledge matched against verbal resources or expectations.” It can be seen from the previous studies that the classification of evidentiality is often concerned with “source of evidence (knowledge)” (e.g., Frawley, 1992, p. 413), or “mode of knowing” (e.g., Willett, 1988), or both (Chafe, 1986, p. 263), but their interaction has seldom been mentioned in the previous studies except for Botne (1997, p.523-524 ); and Squartini (2008, p. 918). Therefore, this paper will argue that the source of evidence and the mode of knowing are inseparable and should be integrated in the study of evidentiality to gain a better understanding of the domain (Squartini, 2008, p. 917).

CDA (Critical Discourse Analysis) aims to reveal “traces of ideological bias” in various discourse contexts (Widdowson, 2007, p. 71). However, ideological bias can often be disguised as facts or common knowledge by the speakers with the help of various linguistic strategies including evidentiality. However, the role of evidentiality relating to persuasive strategies has largely been ignored in previous studies, and therefore deserves attention in this paper.

This paper argues that different types of evidentials can be adopted to examine the underlying ideology of the speaker during the process of communication by addressing their pragmatic functions. It aims to demonstrate how evidentiality can be treated as strategic tools in English diplomatic discourse. In particular, two research questions will be discussed in this paper: How can English evidentiality be classified according to “information sources” and “modes of knowing”? What functions do different types of evidentials have in the context of diplomatic discourse?

The data of this paper include three cases of English diplomatic speeches^2^ from three US presidents: George W. Bush, Barack Obama, and Donald Trump. Each case consists of a corpus of 10 speeches (around 32,500 words) from the same politician, with topics focusing on diplomatic relations and foreign policies. The reason why the author chooses the diplomatic speeches of the three US presidents is that these speakers have been recognized as among the most influential or controversial political speakers. Therefore, it would be interesting to investigate how these political speakers persuade or manipulate their audiences through evidentiality.

This paper takes a data-based approach in retrieving evidential markers manually from the data by three independent annotaters.^3^ These markers are then annotated with innovative codes of nine types of evidentials (see “An Analytical Framework of English Evidentiality” for details). After that, the annotated data are used to do both qualitative and quantitative analysis.

In what follows, we will first provide a brief review of previous studies about evidentiality in discourse studies, particularly focusing on its classifications and functions. Then we will introduce our framework of studying English evidentiality, with most examples from the annotated data on diplomatic discourse. We will examine how evidentials function in maintaining or shaping foreign relations. Finally, we will draw the conclusions and discuss the possible application of this framework for relevant studies.

## Background: Evidentiality in discourse studies

Evidentiality is often categorized by its reliability or subjectivity (e.g., Hart, 2011; Marín-Arrese, 2011b), and treated as a tool of rhetorical persuasion (Antaki and L, 2001, p. 468) or a device of manipulation (e.g., Mushin, 2000; Berlin and Prieto-Mendoza, 2014) in discourse studies. It is even more prominent in strategic discourse including diplomatic discourse, because hearers can easily identify the reliability or validity of evidence, even though they do not trust the speakers at all (Sperber, 2006, p. 184).

For example, Bednarek (2006) observes the role of evidentiality as “epistemological positioning” and proposes four types of evidence in terms of knowledge in media discourse: PROOF, PERCEPTION, PUBLIC KNOWLEDGE, and OBVIOUSNESS. However, her classification has semantic overlapping, especially in “PROOF and OBVIOUSNESS,” which both involve in the forms of evidence concerning with experience (Marín-Arrese, 2011b, p. 792).

Based on Bednarek’s work (2006), Hart (2011, p. 760) associates six categories of evidence, by adding two categories, including EXPERT KNOWLEDGE and EPISTEMIC COMMITMENT, according to the reliability of evidence and the degree of subjectivity. Furthermore, Hart (2010, 2011) treats evidentiality as a legitimizing strategy of “objectification” in immigration discourse. Objectification refers to a legitimizing device by using evidence (evidentials) to manifest its source and reliability to display its objective stance (Hart, 2010, p. 173).

Marín-Arrese (2011b) also investigates the legitimizing strategies from the perspective of cognitive linguistics by analyzing evidentiality and epistemic modality in diplomatic discourse. She proposes that the speakers have an impact on the addressees’ “exercise of epistemic vigilance” by using epistemic positioning strategies, therefore the addressees will accept the speakers’ propositions as true (Marín-Arrese, 2011b, p. 791). Her classification of evidentials (including personal and mediated evidentiality) is concerned with evaluating their validity and (inter)subjectivity (Marín-Arrese, 2011b, p. 793).

Hart (2011) and Marín-Arrese’s (2011b) work provides a sufficient starting point for studying the reliability/validity and (inter)subjectivity of evidence. This paper holds that epistemic modality and evidentiality fall into two different semantic or grammatical domains (see De Haan, 1999; Aikhenvald, 2004; Hart, 2010), because evidentiality focuses on the source and the validity of the evidence, while epistemic modality involves with the speakers’ justification or evaluation toward the evidence.

In addition to the functions of legitimation (persuasion) and stance-taking, evidentiality has also been studied from the perspective of manipulation. Mushin (2000, p. 927), for example, discusses the deictic function of evidentiality in discourse and demonstrates how the speakers manipulate different evidentials to manifest information or knowledge from various perspectives. In particular, Berlin and Prieto-Mendoza (2014) observe that evidentials can be treated as a strategic device of manipulation in political discourse.

Though the above previous studies bring many valuable insights for this study, they failed to provide a clear and workable analytical framework of evidentiality. Therefore, this study will work out a new classification of English evidentiality so as to analyze evidentiality from a new perspective.

## An analytical framework of English evidentiality

As mentioned in the introduction, the previous studies often use “source of knowledge” or “mode of knowing” (or “forms of access to the information”) in the classification of evidentiality. These two notions may cause confusion and misunderstanding to this domain. It is therefore necessary to clarify their relations before proposing a new classification of evidentiality. This paper argues that these two notions are inseparable in classifying evidentials as they are two aspects of the same body. For example, the evidential “I see” not only indicates its mode of knowing as “direct perception” but also marks the speaker’s high commitment to the source of evidence (treated as “personal sources” here). Similarly, the evidential “it is said that” indicates both its mode of knowing as “hearsay” and its source as “the speaker’s low commitment” (treated as “other sources” here). However, the evidential “we know” indicates both its mode of knowing as “indirect evidence assumed from common knowledge” and its source as “shared by the speaker and his/her addressees” (treated as “shared sources” here).

Based on “speaker’s commitment^4^ to information sources” and modes of knowing (Squartini, 2008, p. 918), we put forward a new classification of English evidentiality integrating these two notions. The evidentials indicating sources of information can be divided into three types as “personal sources,” “shared sources,” or “other sources,” depending on speaker’s commitment to the information source. The distinction of “personal sources” and “other sources” lies in whether the speakers take high or low responsibility for information sources, while shared source refers to the information or knowledge assumed to be shared by both the speakers and their addressees.

The trichotomy of sources of evidence is inspired by notions of subjectivity, objectivity and intersubjectivity.^5^ The evidentials from “personal sources” indicate the explicitness of the conceptualizer, which means that the speaker is willing to make full commitment to the source of information. However, evidence of “‘personal sources” is often only known to the speaker himself or herself, which is hard to get attested for the addressees, so it is more subjective. The evidentials from “other sources^6^,” on the other hand, indicate the implicitness of the conceptualizer, which means the speaker intends to “stand back” and let the evidence “speak for itself” (Hart, 2010, p. 173), they are therefore more objective (Marín-Arrese, 2011a, p. 214). As to evidentials from “other sources,” the speaker stands back and makes low commitment to its reliability, just as Aikhenvald (2004, p. 136) claims that “when you might want to distance yourself, when you do not want to take full responsibility for your words,” you may use a quotative or reported evidential. The evidentials from “shared source” mark the information potentially shared by others (intersubjectivity; Nuyts, 2001) and generally assumed as common knowledge or facts by the speaker.

Based on the theories of “direct and indirect evidence” (Willett, 1988, p. 57; Hart, 2011, p. 758), “personal evidentiality” (Marín-Arrese, 2011b, p. 793), and the notions of INFERENCE, ASSUMPTION, and QUOTATIVE (Aikhenvald, 2004, p. 367), and according to the theory of “mode of knowing,” each source can be classified into three categories: direct evidentials, inferential evidentials, and assumed evidentials. Therefore, based on the interplay of “speaker’s commitment to information sources” and “modes of knowing,” the evidentials can be further classified into nine categories:

1. **Personal perceptual evidentials (P.P.)**, indicating evidence from sources based on personal sensory perception (Willett, 1988, p. 57) (e.g., I’ve seen; I saw).
2. **Personal inferential evidentials (P.I.)**, indicating evidence from sources based on personal mental (metaphorical) perception (Greenbaum, 1969, p. 205) or INFERENCE based on visible or tangible evidence (Aikhenvald, 2004, p. 367) (e.g., I can see; it seems to me; it is clear to me) or logical reasoning (Willett, 1988, p. 57; Hart, 2011, p. 758) (e.g., I realize; I’m convinced).
3. **Personal assumed evidentials (P.A.)**, indicating evidence from sources based on assumed personal knowledge or belief (Marín-Arrese, 2011b, p. 793) (e.g., I know; I believe; I think)
4. **Shared perceptual evidentials (S.P.)**, indicating evidence from sources based on shared sensory perception (e.g., you have heard; we have seen).
5. **Shared inferential evidentials (S.I.)**, indicating evidence inferred from shared mental (metaphorical) perception (Greenbaum, 1969, p. 205) or “INFERENCE based on visible or tangible evidence” (Aikhenvald, 2004, p. 367) (e.g., we are seeing; we saw; clearly; it was clear that; it seems that) or logical reasoning (Willett, 1988, p. 57; Hart, 2011, p. 758) (e.g., we realize; we are convinced).
6. **Shared assumed evidentials (S.A.)**, indicating evidence from sources based on general knowledge (Aikhenvald, 2004, p. 367) (e.g., we know; you know; everyone knows).
7. **Quotative evidentials (Q.E.)**, indicating evidence that clearly refers to the source cited (Aikhenvald, 2004, p. 367) (e.g., says; saying; said).
8. **Other inferential evidentials (O.I.)**, indicating evidence inferred from reports or results (Willett, 1988, p. 57; Hart, 2011, p. 758; Marín-Arrese, 2015, p. 213) (e.g., the report indicates; the figure reveals).
9. **Hearsay evidentials (H.E.)**, indicating “reported information with no reference to whom it was” (Aikhenvald, 2004, p. 367) (e.g., some people think; it is said that).

**Figure 1:**
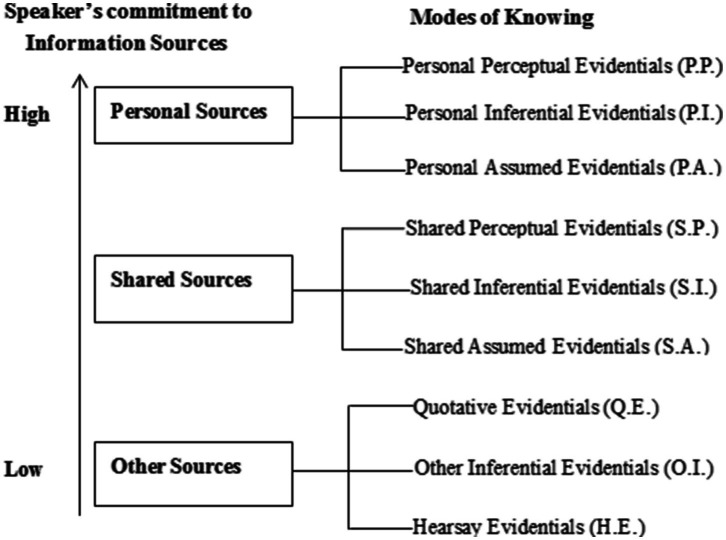
An analytical framework of English evidentiality.

The analytical framework of evidentiality can be illustrated briefly in Figure 1, see the following section for the annotated examples and their pragmatic functions in the context of diplomatic discourse.

“Strength of evidence” is used to assess the reliability or persuasiveness of evidentials. It is concerned with “the extent of validity” of evidentials, which represents the differences between various sources of evidence or modes of knowing (Hart, 2011).

The strength of evidence can be expressed by distance, measured as the relative distance from the center of certainty or truth. In other words, weak evidence means far from certainty or truth. For example, for perceptual categories, the marker “I’ve seen” is stronger in evidence strength than “I’ve heard” because the former is closer to certainty or truth. Again, the latter is more reliable than “I feel.” Evidence from reports is more reliable than anecdotal evidence from other sources. In other words, the weaker elements are less certain and therefore farther from the center of truth. However, it is dangerous to associate strength of evidence to its source type (Fitneva, 2001, p.404) or mode of knowing, though in general the reliability of the first category in each source is stronger than that of the second one. We need to take its “context-specific grammatical formatting and semantics” into consideration (Reber, 2014, p. 357).

## Pragmatic functions of evidentiality in diplomatic discourse

As mentioned above, evidentiality has been studied as legitimization strategies (Hart, 2011; Marín-Arrese, 2011b) or as a tool of manipulation in discourse studies (Mushin, 2000; Berlin and Prieto-Mendoza, 2014). However, it remains unclear about which type of evidentials can be used to persuade or manipulate the addressees and the ideological bias hidden in those evidentials in diplomatic discourse.

Hart (2014, p. 3) proposed that language influences social behaviors and relationships in two key ways: one is the way of normalizing ideology; another is the way of legitimizing action. In other words, normalizing ideology and legitimizing actions may provide a tool for the speaker to influence the addressees’ social behaviors and social relations in specific discourse contexts. However, evidentiality has never been studied from the perspective of the “normalization^7^” of ideology.

In what follows, we will investigate the nine types of evidentials from the perspective of pragmatic functions as effecting foreign relations and actions through legitimation and “normalization” of ideology, as well as uncovering the speakers’ ideological bias and their intentions.

### Evidentials from personal sources

Personal evidentials indicate that the speaker is solely or highly responsible for the information provided, because the evidence is derived from the speaker’s own experience or knowledge. This type of evidence is more subjective than the other two because it is difficult for the audience to verify the source of their information. In the following, several typical examples of personal sources of evidentials will be illustrated to investigate their discourse functions in diplomatic discourse. This type of evidentials has three subcategories: Personal Perceptual Evidentials (P.P.), Personal Inferential Evidentials (P.I.), and Personal Assumed Evidentials (P.A.).

#### Personal perceptual evidentials

Personal Perceptual Evidentials indicate evidentials that come from direct evidence of the speaker’s self-perception. Such evidentials indicate direct evidence and more reliable than the other two types of personal sources, but they are rarely used in the corpus of diplomatic speeches.

For examples:

1. *I* also *saw* < Personal / P.P. > that President Trump had also put my story on the Instagram, so I cried again. (Trump; 2 February 2018)^8^
2. ***I often hear*** < Personal / P.P. >it said that we need moral clarity in this fight. And the suggestion is somehow that if I would simply say, these are all Islamic terrorists, then we would actually have solved the problem by now, apparently < Shared / S.I. >. (Obama; 3 February 2016)

In Example (1), one of North Korean Defectors mentioned that he cried again when he saw President Trump had put his story on the Instagram when he was interviewed at the meeting. By using the Personal Perceptual Evidential “**I saw**,” the speaker provided the direct visual evidence relating to Trump. It is a typical way for describing one’s experience, though no such evidentials (personal visual evidentials such as **I see** or **I saw**) are found in our data in the diplomatic context. As can be seen in the data, the politicians prefer to use shared visual evidentials such as “**We have seen**” rather than personal visual evidentials.

The evidential “**I hear**” In Example (2), however, indicates that the opinion “we need moral clarity in this fight” is not Obama’s own idea. Obviously, this type of evidentials helps the speaker explain the reason why he would do so. In this way, Obama legitimizes his action of war and eases the diplomatic tensions with Islamic countries. Moreover, the strength of this type of evidentials is much weaker than the one in Example (1) as auditory or tactile perception is less dependable than visual perception (Sweetser, 1984, p13).

#### Personal inferential evidentials

According to Greenbaum (1969, p. 205) and Bednarek (2006, p. 640), perceptions can be classified into two categories: **sensory perception** (e.g., I saw; I’ve heard) and **mental perception** or **inference** (e.g., obviously, clearly, apparently, it seems).

However, in our corpus, we discovered that most sensory perceptual evidentials are used in a metaphorical way (see examples in Section “Shared inferential evidentials” for details), which are equivalent to the mental perception or inference described by Greenbaum (1969, p. 205) and Bednarek (2006, p. 640). Thus, we reclassify this kind of evidentials as Personal Inferential Evidentials, which indicate evidence from sources based on personal mental (metaphorical) perception or INFERENCE based on visible or tangible evidence (Aikhenvald, 2004, p. 367). This category also includes inferential evidence based on logical reasoning (e.g., I realize; I’m convinced), which implies that there is certain evidence in the context. Unfortunately, we did not identify any examples of visual perception used as personal mental perception in the data, except for those inferential evidence based on logical reasoning.

For examples:

3. Again **I saw** < Personal/P.I. > that under the sun the race is not to the swift, nor the battle to the strong, nor bread to the wise, nor riches to the intelligent, nor favor to those with knowledge, but time and chance happen to them all. (Cited from the *Bible*)
4. Some have argued we should wait— and that is an option. In my view, it is the riskiest of all options, because the longer we wait, the stronger and bolder Saddam Hussein will become. We could wait and hope that Saddam does not give weapons to terrorists, or develop a nuclear weapon to blackmail the world. But ***I’m convinced*** < Personal / P.I. > that is a hope against all evidence. (Bush; 7 October 2002)

As shown in Example (3), the visual perception “**I saw**” is used metaphorically or rhetorically in which the speaker’s own opinion or stance is implanted. It is equivalent to “I see this fact metaphorically,” and it reflects the speaker’s own ideology.

Similarly, the evidential “**I’m convinced**” used in Example (4) implies that the reason why Bush would hold the opinion is that he was convinced by the evidence comes from his logical reasoning, which has been shown in his previous arguments in the example and in the context of his speech on “Iraq’s threat.” However, we know that most of Bush’s arguments in the speech can be treated as a legitimizing strategy in terms of legitimation by construing a “threat” to “the self” according to the theory of “proximization” (Hart, 2014, p. 167; also see Cap, 2008, 2013). The evidential “**I’m convinced**” also implies that there are tangible evidence in the context, so it can also be seen as a linguistic strategy of persuasion.

#### Personal assumed evidentials

Personal Assumed Evidentials indicate that the information provided is assumed based on one’s own beliefs, knowledge, or thoughts. Generally, those evidence derived from one’s beliefs or thoughts is less dependable than those from knowledge (see Chafe, 1986, p. 263; Marín-Arrese, 2011b, p. 794). For examples:

5. ***I know*** < Personal / P.A. > that many of the issues that I’ve talked about lack the drama of the past. And ***I know*** < Personal / P.A. > that part of Cuba’s identity is its pride in being a small island nation that **could** stand up for its rights, and shake the world. But ***I also know*** < Personal / P.A. > that Cuba **will** always stand out because of the talent, hard work, and pride of the Cuban people. (Obama; 22 March 2016)
6. The Palestinian Authority has rejected your offer at hand, and trafficked with terrorists. You have a right to a normal life; you have a right to security; and *I **deeply believe that*** < Personal / P.A. > you need a reformed, responsible Palestinian partner to achieve that security. (Bush; 24 June 2002)

The triple repetition of the evidential “**I know**” in example (5) means that the speaker has solid evidence in his mind about the information he provides. That is because “**know**” refers to the cognitive process of knowing the truth. Therefore, this kind of premise is very common in the diplomatic speeches of politicians, which can persuade and manipulate the audience by presupposing a certain point of view or stance as fact. However, one cannot see or testify the speaker’s knowledge, so the modal marker can only be seen as subjective modality. In fact, this knowledge reflects speaker’s ideology (*cf*. Afzaal et al., 2022) and foreign policy about Cuba. By adopting three evidentials “**I know**,” Obama expresses his willingness to reshape or restore the political relationship between US and Cuba. Quantitative studies show that Obama used this marker 17 times, compared with five times for Bush and three times for Trump.

The evidential “**I deeply believe**” in Example (6) indicates the evidence comes from the speaker’s thoughts, beliefs or opinions (Chafe, 1986, p. 264). This evidential is seen as more subjective and less reliable than the evidentials in Example (5). Therefore, the proposal “*you need …… that security*” is based on Bush’s own thoughts, which encodes his ideological bias that “Palestinian government is not responsible and needs to reform.” In other words, Bush normalizes his idea about Palestinian government by using the evidential “**I deeply believe**.”

In sum, despite of the different reliability of evidentials, all the personal evidentials show the speakers’ high commitment to their information sources, which may help the speaker to establish an identity as a “responsible” or “authoritative” leader, particularly in the case of Obama.^9^

### Evidentials from shared source

Evidentials from shared source often presuppose common knowledge or truth shared by the speaker and the addressees (Hart, 2010, p. 95). They can evoke collective responsibility between the speaker and the addressees (Marín-Arrese, 2011b, p. 794) which are often adopted to legitimize the speaker’s position or viewpoint from the perspective of intersubjectivity. Besides, evidentials from shared sources often encode ideology, which “derives from the taken-for-granted assumptions, beliefs and value-systems” shared by social groups (Simpson, 1993, p. 5). In this section, we will investigate the examples from three types of shared evidentials, particularly explaining how they normalize ideology and legitimize diplomatic policies and actions in diplomatic speeches.

#### Shared perceptual evidentials

Shared Perceptual Evidentials usually indicate the evidence based on facts or truth perceived by common senses. Sensory perception is the most direct evidence a person can possess (Whitt, 2011, p. 8), especially “visual and auditory perception” (Palmer, 2001, p. 43), and is therefore generally considered more reliable than indirect evidence.

For examples:

7. We’ve experienced the horror of September the 11th. ***We have seen*** < Shared / S.P. > that those who hate America **are willing to** crash airplanes into buildings full of innocent people. Our enemies would be no less willing, in fact, they would be eager, to use biological or chemical, or a nuclear weapon. (Bush; 7 October 2002)
8. The United States and South Korea have made great progress in reducing our trade imbalance and unleashing new prosperity for both of our countries. And, ***as you heard*** < Shared / S.P. **>**, ***your great ambassador and our great ambassador say*** < Other /Q.E. > we have reduced the number to by about 60 percent, which is pretty — pretty great. (Trump; 30 June 2019)

Example (7) is a typic example of shared perception, and the evidential “**We have seen**” indicates that what follows it is a fact that can be seen by everybody. It is true that we can see the tragedy of 9/11. This evidential, therefore, shows the most reliable information source. However, this evidence which Bush used to legitimize his proposal of “war on Iraq” is inadequate, because the tragedy of 9/11 cannot prove Iraq will be using “biological or chemical, or a nuclear weapon” toward America or Iraq has possessed these weapons. Therefore, this kind of evidentials is a powerful strategy which can be used to persuade or manipulate the addressee in legitimizing the speaker’s proposal.

The evidential “**hear**” is normally less reliable than “**see**” as the former evidence is indirect and the latter one is direct. However, the evidential “**you heard**” in Example (8) has been reinforced by a quotative evidential “**your great ambassador and our great ambassador say**,” which can be attested by the addressees. Therefore, when we evaluate the reliability of information sources in discourse interpretation, we need to take the context into consideration.

#### Shared inferential evidentials

Shared Inferential Evidentials indicate evidence inferred from shared mental (metaphorical) perception (Greenbaum, 1969, p. 205) or the information comes from tangible evidence (Aikhenvald, 2004, p. 367) or logical reasoning. Such evidence suggests that it can be inferred from sensory or reasoning evidence shared by the speaker and the addressees, which is often used as a tool to persuade or manipulate the audience into accepting the speaker’s views as fact or truth, in order to normalize the speaker’s ideology or legitimize his/her proposals or actions. However, because the source of these data elements is unclear, they are less reliable than the previous one.

For examples:

9. ^10^ When we look around this city—so beautiful and ***we see*** < Shared / S.I. >people of all faiths engaged in reverent worship, and schoolchildren learning side-by-side, and men and women lifting up the needy and forgotten, ***we see*** < Shared / S.I. > that God’s promise of healing has brought goodness to so many lives. ***We see*** < Shared / S.I. > that the people of this land had the courage to overcome the oppression and injustice of the past and to live in the freedom God intends for every person on this Earth. (Trump; 23 May 2017)
10. ***It is now clearer than ever*** < Shared / S.I. > that Hezbollah militias are the enemy of a free Lebanon -- and all nations, especially neighbors in the region, have an interest to help the Lebanese people prevail. (Bush; 18 May 2008)

Perceptual evidentials are often found to be used as mental perceptions in our diplomatic discourse corpus. For example, two of “**we see**” in Example (9) are both used metaphorically instead of sensorily, because we cannot see “God’s promise” and “people’s courage” directly. This kind of evidentials actually function as effective linguistic strategies in normalizing the speaker’s ideology as people often regard visual perceptions as facts or reality. Therefore, the audience tend to believe and accept his words. In this case, this type of evidentials is an effective strategic tool for strengthening the diplomatic relations with the United States and Israel.

As shown in Example (10), the evidential marker “**It is now clearer than ever**” can be treated as the inference based on visual or tangible evidence (Aikhenvald, 2004, p. 367), though it is less reliable than the evidentials in Example (9).

#### Shared assumed evidentials

Shared Assumed Evidentials often indicate evidence based on general knowledge (Aikhenvald, 2004, p. 367) shared by both the speaker and the hearers. This type of inferential evidentials has been used much more frequently than the previous two categories as it often presupposes the propositions which follow the evidentials as truth without having to make the sources clear. The evidentials in this category are a typical device used by the politicians to manipulate or persuade the addressees to accept their opinions as common knowledge. But it is often less reliable than the previous two categories since its source and reasoning process are both implicit. For examples:

11. And, as ***we know*** < Shared / S.A. >, in 2011, America hastily and mistakenly withdrew from Iraq. As a result, our hard-won gains slipped back into the hands of terrorist enemies. (Trump; 21 August 2017)
12. We have taken these positions because ***we believe*** < Shared / S.A. > that freedom and self-determination are not unique to one culture. These are not simply American values or Western values—they are universal values. (Obama; 25 September 2012)

The evidential “**we know**” used in Example (11) presupposes the proposition ‘in 2011, America hastily and mistakenly withdrew from Iraq’ as a fact. But actually, “in 2011, America withdrew from Iraq” is a fact, but whether America withdrew from Iraq “*hastily and mistakenly*” is not certain. It is only the speaker’s own political stance, which also encodes his ideology toward the issue of Iraq war. By adopting the evidential “**we know**,” Trump tried to normalize his own ideology, thereby delegitimizing the political action of Obama about the withdrawal from Iraq.

The Shared Assumed Evidential “**we believe**” in Example (12) reflects the beliefs or ideology shared by the speaker and the addressee, which is often less reliable than the evidential “**we know**.”

In sum, shared evidentials are the most typical evidentials used by the speakers to normalize their ideology or legitimize their proposals/actions. For example, Trump tried to delegitimize Obama’s political action by using shared assumed evidentials in Example (11). We learned that the facts or truth can also be manipulated or presupposed by using shared evidentials.

### Evidentials from other sources

Evidentials from other sources usually mark the speaker’s minimum responsibility for the information he/she provides, because the speaker takes a back seat in the process of discourse generation (Marín-Arrese, 2011b, p. 794). Compared with shared source and personal source of evidentials, this kind of evidentials can prove or legitimize the speaker’s viewpoint or position from a more objective perspective. In this section, we will investigate some typical examples which illustrate how the evidentials from other sources can reflect or normalize the speaker’s ideology and legitimize diplomatic actions.

#### Quotative evidentials

Quotative evidentials often indicate evidence which clearly points to the citation source (Aikhenvald, 2004, p. 367). The source of this type of evidence is the most explicit and authoritative (Aikhenvald, 2004) among the nine evidential categories, which is often used to justify or legitimize the speaker’s own propositions/proposals or actions. For examples:

13. If this organization is to have any hope of successfully confronting the challenges before us, it will depend, as ***President Truman said*** < Other /Q.E. > some 70 years ago, on the “independent strength of its members.” (Trump; 19 September, 2017)
14. ***The Bible says*** < Other / Q.E. >, “I have set before you life and death; therefore, choose life.” The time has arrived for everyone in this conflict to choose peace, and hope, and life. (Bush; 24 June 2002)

The quotative evidential “**President Truman said**” in Example (13) is used to justify the speaker’s opinion that the hope of successfully confronting the challenges will depend on the “independent strength of its members.” “**The Bible says**” in Example (14) is used by Bush to legitimize his proposal and normalize his ideology. In the context of this speech, Bush holds that if the Palestinian people want peace, hope, and life, they need to accept his proposal of electing a new leader. Both the evidentials indicate that there are explicit and authoritative sources for the evidence, which can add much credibility for the speakers’ opinions, therefore making the two speakers’ stance more objective.

#### Other inferential evidentials

Other Inferential Evidentials often refer to the linguistic markers indicating “report-based inferences” (Marín-Arrese, 2015, p. 219) or result-based inferences (Willett, 1988, p. 57; Hart, 2011, p. 758). In general, such evidence derived from reports or results is indirect evidence. However, the specific authority and source reliability of such evidentials should be judged according to the context. For examples:

15. But I do not want to put the cart before the horse. We do not have a strategy yet. I think what I’ve seen in **some of the news reports suggests** < Other / O.I. > that folks are getting a little further ahead of where we are at than we currently are. And I think that’s not just my assessment, but the assessment of our military as well. (Obama; 29 August 2014)
16. Today, ***estimates indicate*** < Other / O.I. > that Iran is only 2 or 3 months away from potentially acquiring the raw materials that could be used for a single nuclear bomb. (Obama; 2 April 2015)

The evidential marker “**some of the news reports suggests**” in Example (15) is aimed to provide the evidence for legitimizing the speaker’s own assessment that “*folks are getting a little further ahead of where we are at than we currently are*.” However, the source of the new reports is not quite clear. In the same way, the evidential “estimates indicate” shows that the source of information is the report, which is used to prove Obama’s point of view “*Iran is only two or three months away from potentially acquiring the raw materials that could be used for a single nuclear bomb*” in Example (16).

The evidential in Example (16) indicates lower credibility or weaker strength compared with that in Example (15), since the source of the estimates is implicit. The speaker may adopt it deliberately as a manipulation or persuasion tool to mystify the responsibility of his stance (Marín-Arrese, 2011b, p. 794) or avoid the possible conflicts. Moreover, this type of evidentials clearly helps the speakers in normalizing their own judgments or ideological bias to legitimize their proposals.

#### Hearsay evidentials

Hearsay Evidentials indicate that evidence is transmitted information without clear source (Aikhenvald, 2004, p. 367), which is often considered as “the most prominent form of indirect evidence” (Whitt, 2011, p. 9). The source of the information for this type of evidentials is often unknown or intentionally hidden by the speaker. It is often treated as the least reliable (Whitt, 2011) among the three categories of other source. The most popular expression of this type of evidentials is “it is said that.” For examples:

17. ***Some in China think that*** < Other / H.E. > America will try to contain China’s ambitions; ***some in America think that*** < Other / H.E. > there is something to fear in a rising China. I take a different view. (Obama; 27 July 2009)
18. ***It was said*** < Other / H.E. > during World War I, the Canadians never budge. (Bush;1 December 2004)

The evidentials in examples (17) and (18) are the weakest evidence, because the speaker is not clear about the source of the information, which makes the information they provide less reliable and convincing. Such evidentials indicate that the source of information is intentionally hidden by the speaker during the transmission of the information, making it difficult for the audience to verify the source and authenticity of the information. Interestingly, this kind of evidentials is also a persuasion strategy and is often used to delegitimize other people’s opinions (which are often different from the speaker’s). As in Example (17), Obama explicitly said “I take a different view.” By denying these two opinions from unknown sources, Obama sought to ease the diplomatic tensions between China and the United States.

## Quantitative analysis of evidential categories in diplomatic discourse

### Sources of evidentials

**Figure 2:**
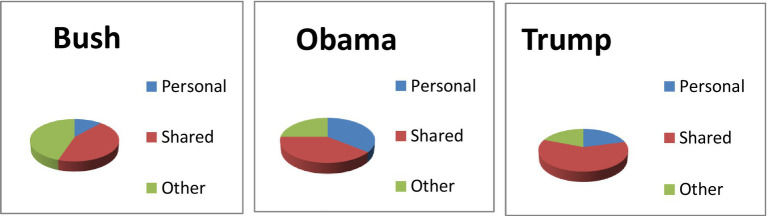
The distribution of sources of evidentials in three cases.

As illustrated in Table 1 and Figure 2, Obama ranks first in the total numbers of evidentials (184 counts) among three speakers, followed by Bush (156 counts) and Trump (110 counts). With respect to the distributions of sources of evidentials, shared evidentials take the first position in the cases of Trump (60%) and Obama (39.13%). However, Bush is the only speaker who used more other evidentials (44.87%) than shared evidentials (43.59%) and personal evidentials (11.54%).

**Table 1:** The distribution of sources of evidentials in three cases.

Speakers	George W. Bush		Barack Obama		Donald Trump	
Sources of evidentials	No.	Percent	No.	Percent	No.	Percent
Personal	18	11.54%	66	35.87%	23	20.91%
Shared	68	43.59%	72	39.13%	66	60%
Other	70	44.87%	46	25%	21	19.09%
Total	156	100%	184	100%	110	100%

**Figure 3:**
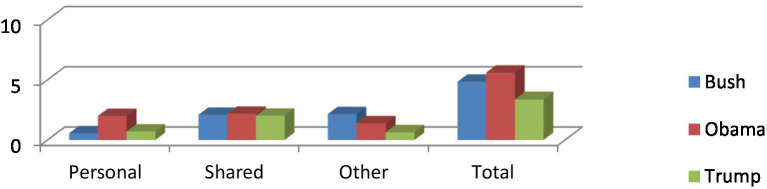
A comparison of sources of evidentials in three cases.

In order to compare the frequencies of evidential types among the three cases, I also worked out the figures of per 1000 words (henceforth, ptw) for each evidential type and their average figures based on the original size of data.^11^ As demonstrated in Table 2 and Figure 3, Obama ranks first in terms of the frequencies of evidential sources both in total (5.6 ptw) and in personal sources (2.01 ptw) and shared sources (2.19 ptw). Following Obama, Bush comes second in the total number of evidentials (4.84 ptw), but he used the most other evidentials (2.17 ptw) among three speakers. It is interesting to see that although Trump used much less other evidentials than Bush and Obama, he adopted more personal evidentials than Bush. Overall, the differences of the total evidentials and the other categories are significant among three speakers except shared evidentials.

**Table 2:** A comparison of sources of evidentials in three cases.

Speakers	George W. Bush		Barack Obama		Donald Trump		*P*-value	SoD.
Sources of evidentials	No.	ptw.	No.	ptw.	No.	ptw.		
Personal	18	0.56	66	2.01	23	0.71	5.672e-09	Yes
Shared	68	2.11	72	2.19	66	2.03	0.9065	No
Other	70	2.17	46	1.40	21	0.65	1.521e-06	Yes
Total	156	4.84	184	5.6	110	3.39	0.000126	Yes

If *p*-value is less than 0.05, then the difference of the relevant category among three speakers is significant.

### Types of evidentials

**Figure 4:**
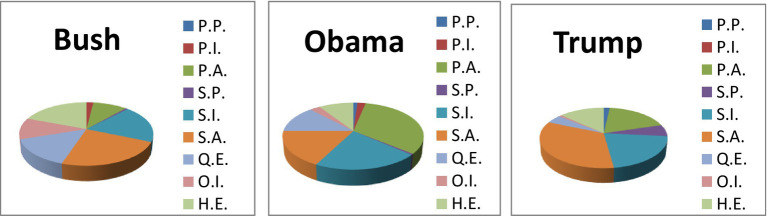
The distribution of types of evidentials in three cases.

In terms of the quantitative analysis of evidential types, it can be seen in Table 3 and Figure 4 that evidentials of S.A. (Shared Assumed Evidentials) and S.I. (Shared Inferential Evidentials) take up the first and second positions in both cases of Trump and Bush. However, in the case of Obama, evidentials of P.A. (Personal Assumed Evidentials) ranks first (32.61%), followed by evidentials of S.I. and S.A. Interestingly, Obama used the least S.P.(Shared Perceptual Evidentials), and P.P. (Personal Perceptual Evidentials) as the same as Bush, while P.I (Personal Inferential Evidentials) and O.I. (Other Inferential Evidentials) are adopted the least in the case of Trump.

**Table 3:** The distribution of types of evidentials in three cases.

Speakers	George W. Bush		Barack Obama		Donald Trump	
Types of evidentials	No.	Percent	No.	Percent	No.	Percent
P.P.	0	0	2	1.09%	2	1.82%
P.I.	3	1.92%	4	2.17%	0	0
P.A.	15	9.62%	60	32.61%	21	19.09%
S.P.	1	0.64%	1	0.54%	4	3.64%
S.I.	30	19.23%	39	21.20%	24	21.82%
S.A.	37	23.72%	32	17.39%	38	34.54%
Q.E.	23	14.74%	24	13.04%	5	4.54%
O.I.	17	10.90%	5	2.72%	1	0.91%
H.E.	30	19.23%	17	9.24%	15	13.64%
Total	156	100%	184	100%	110	100%

**Figure 5:**
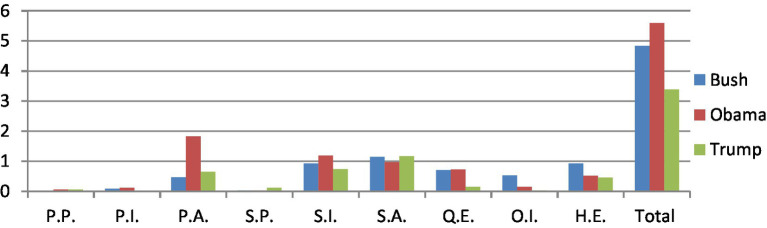
A comparison of types of evidentials in three cases.

As shown in Table 4 and Figure 5, Obama takes up the first position in nearly half of the categories of evidential types in terms of frequencies, including the evidentials of P.I., P.A., S.I. and Q.E., while Bush ranks first in the use of Q.I. and H.E. evidentials among the three speakers. It is surprising to see that Trump adopted far less evidentials in most types compared with the other two speakers, though he used the most S.P. and S.A. evidentials.

**Table 4:** A comparison of types of evidentials in three cases.

Speakers	George W. Bush		Barack Obama		Donald Trump		*P*-value	SoD
Types of evidentials	No.	ptw.	No.	ptw.	No.	ptw.		
P.P.	0	0	2	0.06	2	0.06	0.3725	No
P.I.	3	0.09	4	0.12	0	0	0.1589	No
P.A.	15	0.47	60	1.83	21	0.65	1.303e-08	Yes
S.P.	1	0.03	1	0.03	4	0.12	0.2217	No
S.I.	30	0.93	39	1.19	24	0.74	0.1765	No
S.A.	37	1.15	32	0.97	38	1.17	0.7095	No
Q.E.	23	0.71	24	0.73	5	0.15	0.001409	Yes
O.I.	17	0.53	5	0.15	1	0.03	0.0001022	Yes
H.E.	30	0.93	17	0.52	15	0.46	0.0356	Yes
Total	156	4.84	184	5.6	110	3.39	0.000126	Yes

If *p*-value is less than 0.05, then the difference of the relevant category among three speakers is significant.

Overall, statistical analyses manifest that the differences of most evidential types in frequencies among three cases are not significant except the categories of P.A., Q.E., O.I. and H.E. evidentials.

Evidence from different sources shows that speakers bear different degrees of discourse responsibility for their opinions, and reflect their different ways of persuasion or manipulation. In other words, speakers often use different categories of sources (evidentials) to normalize their ideology and to legitimize their proposals or actions (including the delegitimization of views that are different from their own). For example, according to the quantitative analysis, shared evidentials come first in the corpus of Trump (57.98%) and Obama (38.92%). However, Bush used more evidentials from other sources (70 counts) than shared evidentials (68 counts), but the difference is not significant. The results show that evidentials from shared sources is a typical tool of manipulation in diplomatic discourse since the information provided are often presupposed as facts or truth or general knowledge.

For example, Trump used far more shared evidentials (69 counts) than personal evidentials (29 counts) and other evidentials (21 counts). Take his speech “to the 73rd Session of UN General Assembly” for example (No. 7 speech in Appendix Table 3), Trump used 7 counts evidentials from shared source to legitimize his opinion to persuade his addressees, such as “**we see**,” “**we have seen**,” “**we know**,” and “**we believe**.”

Using more evidential markers from personal sources usually means that the speaker is more willing to take verbal responsibility for his opinion or that he is more confident in his own credibility. For example, Obama used far more personal evidentials than the other two speakers (67 counts vs. Trump, 29 counts and Bush: 18 counts). For instance, in his speech to “Representatives of the African Union” (No. 6 speech in Appendix Table 2), Obama used personal evidentials for 19 counts, including **“I believe**” (13 counts), “**I think**” (2 counts), “**I know**” (1 count), and “**I’m convinced**” (1 count). This means that he is confident in his own words and willing to take the rhetorical responsibility for his point of view. These facts help Obama establish a more reliable and intimate relationship with his audience. This also helps in constructing his identity as an authoritative leader (*cf*. Reber, 2014).

However, evidentials from other sources usually reflect that the speaker is less willing to take responsible for his or her words. This often helps the speaker mystify the responsibility for his/her own stance-taking acts (Marín-Arrese, 2011b, p. 794). It also means that the speaker adopts a more authoritative and objective style of speech. For example, Bush used more other evidentials than the other two politicians (Bush: 70 counts; Obama: 46 counts; Trump 21 counts). In his speech “Address to the United Nations General Assembly in 2002” (Appendix Table 1 No. 3 speech), Bush uses 12 other sources to legitimize his proposal, such as: “**The council said**,” “**The U.N. Commission on Human Rights found**,” “**The Secretary General’s high-level Coordinator for this issue Reported** “and” **the Iraqi Regime said**.” This means that Bush deliberately insulates himself from the responsibility of discourse generation by providing authoritative sources of evidence, thereby making his proposals appear more objective and reliable. These evidentials helped him win public and coalition support for starting the Iraq war.

In addition, there are elements of different sources and intensities which capture the subjectivity and intersubjectivity of the speaker’s positions. According to Portner (2009, p. 131) and Verhulst et al. (2013, p. 211), subjectivity refers to a speaker’s commitment to the power of a claim (see Searle and Vanderveken, 1985). Intersubjectivity refers to the shared commitment of both speaker and listener to the power of a claim. Rather, the difference between subjectivity, intersubjectivity, and objectivity lies in the degree to which the speaker is committed to the power of the claim he is making, which can be judged by different conventions. The scope of subjectivity and intersubjectivity includes “the extent to which the speaker/author is personally responsible and accountable for the information provided (subjectivity), or whether the information is potentially shared by others (intersubjectivity)” (Marín-Arrese, 2011b, p. 794; See Nuyts, 2001). In addition, evidentials from different sources also reveal the speaker’s stance in the way of subjectivity or intersubjectivity. Drawing on Portner (2009, p. 131) and Verhulst et al. (2013, p. 211), subjectivity here refers to speaker’s commitment to the force of an assertion^12^ (see Searle and Vanderveken, 1985), and intersubjectivity refers to the shared commitment of speaker-hearer to the force of an assertion.

**Figure 6:**
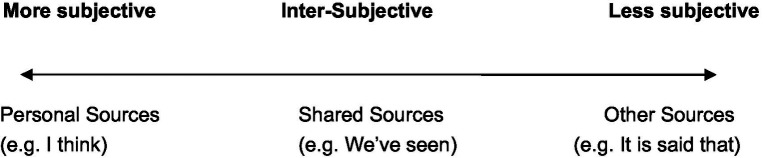
The scale of (inter)subjectivity vs. the source of evidence.

Particularly, different sources of evidentials can differentiate or evaluate the stance of subjectivity, intersubjectivity, and objectivity by analyzing the speaker’s respective responsibility toward his/her assertion. The scale of subjectivity and intersubjectivity involves “the degree to which the speaker/writer assumes personal responsibility and accountability (subjectivity) for the information proffered, or whether the information is presented as potentially shared by others (intersubjectivity)” (Marín-Arrese, 2011b, p. 794; also see Nuyts, 2001). The interaction of sources of evidence and the subjectivity and intersubjectivity can be illustrated as Figure 6.^13^

That is to say, justified by personal evidentials (e.g., **I think**), the speaker’s stance is more subjective as the speaker takes the sole responsibility for his or her proposition, while the stance backed by shared evidentials (e.g., **We’ve seen**) is inter-subjective because the information is assumed to be shared by the speaker and his or her addressees. However, the stance supported by other evidentials (e.g., **It is said that**) is more objective since the verbal responsibility has been deliberately hidden or mystified by the speaker.

## Conclusion

Overall, this paper proposed an analytical framework of evidentiality in English diplomatic discourse by taking both ‘speaker’s commitment to sources of knowledge’ and ‘modes of knowing’ into account. It demonstrated how nine different types of evidentiality function in diplomatic discourse by a data-based approach, especially in terms of speaker commitments and normalization of ideology and (de)legitimation of proposals. It also examined the use of different sources and types of evidentials in three corpuses of English diplomatic speeches.

The most striking results are:

1. According to “the speaker’s commitment to the sources,” evidentiality can be classified into three categories: personal sources; shared sources, and other sources.
2. According to “mode of knowing,” each source can be classified into three categories: direct evidentials, inferential evidentials, and assumed evidentials. Based on the interplay of “speaker’s commitment to information sources” and “modes of knowing,” the evidentials can be further classified into nine categories: Personal Perceptual Evidentials, Personal Inferential Evidentials, Personal Assumed Evidentials, Shared Perceptual Evidentials; Shared Assumed Evidentials; Quotative Evidentials, Other Inferential Evidentials, Hearsay Evidentials.
3. The main functions of evidentiality in diplomatic discourse are: to persuade or to manipulate the addressees; to normalize the speaker’s ideology; to legitimize the speaker’s proposals or actions; to delegitimize other people’s different views.
4. Trump used far more shared sources of evidentials than the other two sources. Shared sources are the most frequently used strategies of persuasion or manipulation in diplomatic discourse, allowing the audience to mistake the speaker’s opinions for facts. Meanwhile, such evidentials reflect the speaker’s ideological bias, because they encode the speaker’s presupposition of authority, facts, or shared knowledge.
5. Obama ranked first in using personal sources, which means that he is more willing to take verbal responsibility for his ideas or he is more confident in his own credibility, which helps him build a closer relationship with the audience.
6. Bush used the most other sources of information, which reflects his lower responsibility for the information he offered. This helps him avoid his verbal responsibility. On the other hand, it means that he has adopted a more objective and authoritative style of speech.
7. Different sources of evidentials can reflect the speakers’ corresponding responsibility for their propositions and reveal their subjective or intersubjective stance.

In sum, the evidential categories proposed in this paper may shed light on exploring the pragmatic functions of evidentiality in various discourse contexts, especially for strategic discourse, such as media discourse, war discourse, racial discourse, courtroom discourse, and religious discourse.

## Data availability statement

The raw data supporting the conclusions of this article will be made available by the authors, without undue reservation.

## Author contributions

The author confirms being the sole contributor of this work and has approved it for publication.

## Funding

The project of National Social Science Fundation of China (No. 17BYY187).

## Conflict of interest

The author declares that the research was conducted in the absence of any commercial or financial relationships that could be construed as a potential conflict of interest.

## Publisher’s note

All claims expressed in this article are solely those of the authors and do not necessarily represent those of their affiliated organizations, or those of the publisher, the editors and the reviewers. Any product that may be evaluated in this article, or claim that may be made by its manufacturer, is not guaranteed or endorsed by the publisher.

## Supplementary material

The Supplementary material for this article can be found online at: https://www.frontiersin.org/articles/10.3389/fpsyg.2022.1019359/full#supplementary-material

## Footnotes

^1^Evidential types refer to the subcategories of evidentiality, which have been studied from various perspectives, such as ‘type of evidence’ (*cf.* Anderson, 1986, p. 274), or ‘source of evidence (knowledge)’ (*cf.* Frawley, 1992, p. 413).

^2^The detailed information of their speeches is shown in the Appendix.

^3^The annotators are the author and her two fellow researchers. Each evidential will be annotated by abbreviations in two aspects:‘speaker’s commitment to information sources’ and ‘modes of knowing’, For example, "I saw" will be annotated as **(P.P.)**, which means **Personal Perceptual Evidentials**. The process of annotation is very complicated. Some controversial evidential markers need group discussion and careful reconsideration.

^4^Speaker’s commitment draws on Marín-Arrese’s idea of "personal responsibility for the communicated proposition" (2011a, p.214).

^5^This idea is inspired from the notions of personal vs. shared responsibility vs. objectivity (Marín-Arrese, 2011a, 2013, p. 424).

^6^"Other sources" refer to the information sources that the speaker stands back and takes no responsibility for their reliability.

^7^"Normalization of ideology" has never been defined before. In this paper, it refers to the process when the speaker uses linguistic strategies (including evidentiality), consciously or unconsciously, to convince his/her addressees that his/her ideological bias hidden in the propositions is normal and should be treated as facts, truth or common knowledge.

^8^Example (1) comes from other sources as there are no such examples in the data.

^9^Obama used far more personal evidentials than the other two speakers.

^10^The Example (9) comes from other sources as there are no examples of ‘shared visual perception used in a metaphorical way’ found in the data.

^11^The total number of words of the data is 97,496 words, with 32,226 words in Bush’s speeches, 32,825 words in Obama’s speeches and 32,445 words in Trump’s speeches, respectively. For details, please refer to the tables in the Appendix.

^12^Here the force of an assertion refers to the illocutionary force which is beyond the content of a proposition. In this paper, it refers to the speaker’s stance towards the assertion, including the modal stance and evidential stance. That’s because modal markers can be part of implicit performatives and carry illocutionary force (also see Boyd and Thorne, 1969).

^13^This scale is inspired by the subjectivity/intersubjectivity scales of Marín-Arrese (2013, p. 429–430).
