# Supplementary material for: Pragmatic functions of evidentiality in diplomatic discourse: Toward a new analytical framework
Source: Front Psychol. 2022 Dec 1;13:1019359. doi: 10.3389/fpsyg.2022.1019359 (PMC9751786; doi:10.3389/fpsyg.2022.1019359)
Supplement: Supplementary file 1 [file Data_Sheet_1.docx]

**Appendix**

Table 1 George W. Bush’s speeches^^[[1]](#footnote-1)^^

| NO. | Time | Place | Theme/Title | Listeners | Number of words |
| --- | --- | --- | --- | --- | --- |
| 1 | 10 November 2001 | U.N.  New York | 2001 UN General Assembly Address | participants of 2001 UN General Assembly | 2502 |
| 2 | 24 June 2002 | Rose Garden, Washington, D.C. | Speech on Israel­Palestine Two­State Solution | American people | 1882 |
| 3 | 12 September 2002 | U.N.  New York | 2002 UN General Assembly Address | participants of 2002 UN General Assembly | 2741 |
| 4 | 7 October 2002 | Cincinnati  Ohio | Address on the Threat of Iraq | American people | 3367 |
| 5 | 21 September 2004, | U.N.  New York | 2004 UN General Assembly Address | participants of 2004 UN General Assembly | 3058 |
| 6 | 1 December 2004 | Halifax, Nova Scotia, Canada | Address on U.S. and Canada Relations and the War on Terrorism | Prime Minister and other officials of Canadian Government | 3154 |
| 7 | 30 November 2005 | U.S. Naval Academy, Annapolis, Maryland | Iraq Strategy Address | members of the U.S. Naval Academy | 5653 |
| 8 | 13 January 2008 | Abu Dhabi, United Arab Emirates | Address on Freedom and Extremism in the Middle East | People of United Arab Emirates | 3235 |
| 9 | 19 March 2008, | The Pentagon, Arlington Virginia | Address on The War in Iraq After 5 Years | Members of the Defense Department | 3228 |
| 10 | 18 May 2008 | Sharm el Sheikh, Egypt | World Economic Forum Address | participants of World Economic Forum | 3406 |
| Total number of words | | | | | 32226 |

Table 2 Barack Obama’s speeches^^[[2]](#footnote-2)^^

| NO. | Time | Place | Theme/Title | Listeners | Number of words |
| --- | --- | --- | --- | --- | --- |
| 1 | 27 July 2009 | The White House | Remarks on U.S.-China Relations | President Hu of China | 2419 |
| 2 | 18 March 2011 | The White House | Address on Libya | American people | 1265 |
| 3 | 25 May 2011 | Westminster Hall, London | Speech to the British Parliament | Members of the British Parliament | 4269 |
| 4 | 25 September 2012 | U.N.  New York | 2012 UN General Assembly Address | participants of 2012 UN General Assembly | 4060 |
| 5 | 2 April 2015 | Rose Garden, Washington, D.C | Statement on Iran Nuclear Agreement | American people | 2587 |
| 6 | 28 July 2015 | African Union Headquarters, Addis Ababa, Ethiopia | Speech to Representatives of the African Union | Representatives of the African Union | 6023 |
| 7 | 15 October 2015 | The White House | Statement on Afghanistan | The American people | 2447 |
| 8 | 13 November 2015 | Washington, D.C | Address on the Terrorist Attacks in Paris | The American people | 577 |
| 9 | 3 February 2016 | Baltimore, Maryland | Islamic Society of Baltimore Address | Members of the Islamic Society of Baltimore | 5092 |
| 10 | 22 March 2016 | Havana, Cuba | Address to the People of Cuba | People of Cuba | 4086 |
| Total number of words | | | | | 32825 |

Table 3 Donald Trump’s speeches^^[[3]](#footnote-3)^^

| NO. | Time | Place | Theme/Title | Listeners | Number of words |
| --- | --- | --- | --- | --- | --- |
| 1 | 21 May  2017 | Riyadh | Speech to the Arab Islamic American Summit | participants of the Arab Islamic American Summit | 3415 |
| 2 | 21 August 2017 | Fort Myer  Arlington, Virginia | Remarks on the Strategy in Afghanistan and South Asia | The cabinet members and American military people | 2949 |
| 3 | 19 September 2017 | U.N.  New York | Remarks to the 72nd Session of UN General Assembly | participants of 2017 UN General Assembly | 4594 |
| 4 | 13 October 2017 | The White House | Remarks on Iran Strategy | The American people | 1984 |
| 5 | 10 November 2017 | Da Nang, Vietnam | Remarks at APEC CEO Summit | the people and business leaders of the Indo-Pacific region | 3438 |
| 6 | 26 January 2018 | Davos, Switzerland | Remarks to the World Economic Forum | participants of the World Economic Forum | 2781 |
| 7 | 25 September 2018 | U.N.  New York | Remarks to the 73rd Session of UN General Assembly | participants of 2018 UN General Assembly | 3642 |
| 8 | 30 June 2019 | Seoul, Republic of Korea | Remarks to Korean Business Leaders | Korean Business Leaders | 3881 |
| 9 | 24 September 2019 | U.N.  New York | Remarks to the 74th Session of UN General Assembly | participants of 2019 UN General Assembly | 3857 |
| 10 | 23 October 2019 | The White House | Remarks on the Situation in Northern Syria | The American people | 1904 |
| Total number of words | | | | | 32445 |

1. All of George W. Bush’s speeches listed here can be found at the following website: <https://americanrhetoric.com/gwbushspeeches.htm>, [accessed on August 18^th^, 2019]. [↑](#footnote-ref-1)
2. All of Barack Obama’s speeches listed here can be found at the following two websites: <http://www.whitehouse.gov/briefing-room/speeches-and-remarks>, [accessed on April 8^th^, 2012] and

   <https://americanrhetoric.com/barackobamaspeeches.htm>, [accessed on August 20^th^, 2019]. [↑](#footnote-ref-2)
3. All of Donald Trump’s speeches listed here can be found at the following website: <https://www.whitehouse.gov/issues/foreign-policy/>, [accessed on October 25^th^, 2019]. [↑](#footnote-ref-3)
